# An App for Classifying Personal Mental Illness at Workplace Using Fit Statistics and Convolutional Neural Networks: Survey-Based Quantitative Study

**DOI:** 10.2196/17857

**Published:** 2020-07-31

**Authors:** Yu-Hua Yan, Tsair-Wei Chien, Yu-Tsen Yeh, Willy Chou, Shu-Chen Hsing

**Affiliations:** 1 Superintendent Office Tainan Municipal Hospital (Managed by Show Chwan Medical Care Corporation) Tainan Taiwan; 2 Department of Hospital and Health Care Management Chia Nan University of Pharmacy and Science Tainan Taiwan; 3 Department of Medical Research Chi Mei Medical Center Tainan Taiwan; 4 Medical School St George’s, University of London London United Kingdom; 5 Department of Physical Medicine and Rehabilitation Chung Shan Medical University Taichung Taiwan; 6 Department of Physical Medicine and Rehabilitation Chiali Chi Mei Hospital Tainan Taiwan; 7 Respiratory Therapy Unit Chi Mei Medical Center Tainan Taiwan

**Keywords:** respiratory therapist, ELMI app, Rasch analysis, convolutional neural network, mental health, mobile phone

## Abstract

**Background:**

Mental illness (MI) is common among those who work in health care settings. Whether MI is related to employees’ mental status at work is yet to be determined. An MI app is developed and proposed to help employees assess their mental status in the hope of detecting MI at an earlier stage.

**Objective:**

This study aims to build a model using convolutional neural networks (CNNs) and fit statistics based on 2 aspects of measures and outfit mean square errors for the automatic detection and classification of personal MI at the workplace using the emotional labor and mental health (ELMH) questionnaire, so as to equip the staff in assessing and understanding their own mental status with an app on their mobile device.

**Methods:**

We recruited 352 respiratory therapists (RTs) working in Taiwan medical centers and regional hospitals to fill out the 44-item ELMH questionnaire in March 2019. The exploratory factor analysis (EFA), Rasch analysis, and CNN were used as unsupervised and supervised learnings for (1) dividing RTs into 4 classes (ie, MI, false MI, health, and false health) and (2) building an ELMH predictive model to estimate 108 parameters of the CNN model. We calculated the prediction accuracy rate and created an app for classifying MI for RTs at the workplace as a web-based assessment.

**Results:**

We observed that (1) 8 domains in ELMH were retained by EFA, (2) 4 types of mental health (n=6, 63, 265, and 18 located in 4 quadrants) were classified using the Rasch analysis, (3) the 44-item model yields a higher accuracy rate (0.92), and (4) an MI app available for RTs predicting MI was successfully developed and demonstrated in this study.

**Conclusions:**

The 44-item model with 108 parameters was estimated by using CNN to improve the accuracy of mental health for RTs. An MI app developed to help RTs self-detect work-related MI at an early stage should be made more available and viable in the future.

## Introduction

### Background

Mental illness (MI) is common among those who have come in contact with it at the workplace [1] and globally account for 32.4% of years living with a disability [2]. An estimated 264 million people suffer from depression, with many of these people having symptoms of anxiety [3]. The lost productivity cost for depression and anxiety disorders adds up to 1 trillion US dollars globally each year [3]. Not only are absence and the direct costs harmful to organizations but the effects are also concerned with workers who suffer from MI and still remain on the job. Accordingly, employers are increasingly paying attention to presenteeism—decreased productivity because of health problems—among employees who remain present at work [4]. This is because presenteeism might result in a higher economic cost than absenteeism or the medical costs paid by employers [5]. It is worth investing in mental health promotion and prevention (MHPP) programs for employees in the workplace. Every dollar invested in stepping up treatment (or prevention) for common MIs (eg, depression and anxiety) leads to a fourfold return in better health and the passion or the capability to work [6].

### An App Required to Assess MI

Although there has been an increase in MHPP programs globally in recent years, only 7% of such initiatives are carried out at the workplace [1]. In 2015, a Cochrane systematic review [7] evaluated evidence on the effectiveness of interventions to prevent occupational stress in health care workers [8]. Most of them were restricted to measuring work-related stress and/or burnout by using validated tools, particularly lacking the classifications, such as true (or false) health (or illness). As such, a novel application of a more holistic assessment of workplace mental health, including a broader scope of mental health outcomes (eg, the classification of response patterns in visual displays), is required to explore the potential benefits that improve effective classifications of true or false (ie, with less confidence) MI.

### MI App at Workplace

Mobile health interventions (ie, MI apps) are used to monitor mental health and are an increasingly popular approach available for both individuals and organizations [9]. However, at present, there is a lack of research on the effectiveness of mobile MI apps in pattern classifications for smartphone users. This is because many assessment tools are merely focused on the strata of mental health [10-12] instead of the classifications of response patterns (eg, answering questions carelessly, cheating, or guessing) [13] to verify the respondents’ MI classification (false or true with confidence).

Although numerous studies have been conducted on MI apps [9,14-18], few have used the algorithm of artificial neural networks (ANNs) in classifying MI, particularly with convolutional neural networks (CNNs). Traditionally, cutoff scores are often used in the classification of MI [19,20].

Most of the classifications were based on the extent (or, say, strata) to which MI or other disorders are determined by summation scores or equivalent measures of a scale (eg, patients with a psychotic disorder [21] or those with 2 or 3 groups of nurse burnouts [or bullied victims] at the workplace [19,20]). To the best of our knowledge, none of the studies have applied the response pattern to classify the features of MI (or other disorders) with CNN modules to highlight less confident cases of MI characterized by the aberrant response pattern that deviated from normal cases.

### Targeted Health Care Workers

Although we note that mental health has been a concern in physicians [22] and nurses [1], other health care professionals and hospital staff should also be looked after considering the similar working environment to physicians and nurses and the nature of responsibilities that put them at great risk of MI. Thus, it is worth studying how workplace-based organizational interventions can improve the mental health and well-being of health care workers, including respiratory therapists (RTs) who played a vital role in the frontline care of patients with COVID-19 in 2020.

### Study Objectives

This study aimed to (1) determine featured variables used for CNN in the classification of MI, (2) differentiate MI patterns endorsed by participants, and (3) design an MI app for smartphones as a web-based assessment.

## Methods

### Data Source

A survey-based quantitative study was conducted to invite RTs in Taiwan hospitals to answer questions about MI at the workplace. A total of 107 institutes (ie, 88 regional hospitals and 19 medical centers) were targeted according to the hospital list of Taiwan National Health Insurance Administration, and 1521 (753 and 768 in regional hospitals and medical centers, respectively) RTs who were registered in the Taiwan Society for Respiratory Care in January 2019 were included in the study.

If the confidence level and the intervals were set at 0.05 and plus or minus 5%, respectively, and applied to the population of 1521 registered RTs, 307 are required for the sample size [23,24]. We estimated that the percentage of candidates’ refusal to respond to the survey was 40%. Therefore, the minimal number of the study sample size was 530 (n=307/[1−0.4]).

We delivered 6 copies of the 44-item questionnaire (ie, including 2 sets of the emotional labor and mental health [ELMH] questionnaire [25,26]; Multimedia Appendix 1) to 107 targeted workplaces in hospitals. A total of 642 (6×107) RTs with at least three months of experience working in the hospital were randomly selected and invited to complete the ELMH survey in March 2019.

Taking into consideration patient rights, interest, and privacy, an informed consent form was included in the mail to each hospital. Candidates were allowed to decline to answer the anonymous questionnaire. The questionnaire was asked to be mailed back to our study clerk in an enclosed envelope. A total of 352 (>307 required sample designed in the first paragraph) questionnaires were eligible, with a return rate of 54.8% (352/642). Data were deposited in Multimedia Appendix 2.

This study was monitored by the institutional review board of Show Chwan Memorial Hospital with the approval ID number (1080105) before data collection. All hospital and study participant identifiers were stripped.

### Featured Variables and Factor Domains With Unsupervised Learning

Unsupervised learning indicates agnostic aggregation of unlabeled data sets yielding groups or clusters of entities with shared similarities that may be unknown before the analysis step [27,28]. Featured variables consist of 44 items including the 24-item ELMH subscales. Exploratory factor analysis (EFA) was applied to determine factors retained in the study based on the eigenvalue ≥1.0. Factor scores with the Bartlett approach [29] to yield weighted domain scores for each factor (ie, domain) were analyzed to obtain personal measures and fit statistics using the Rasch model with continuous responses [30-32].

Next, participants were plotted to cluster classes based on the criteria of outfit mean square errors (MNSQs; cutting at 2.0 [33]) and measures at zero logits (ie, log odds) in 4 quadrants on axes x and y, respectively.

The definition of measure in logit and outfit MNSQ is worth mentioning. The former is analogical to the traditional summation score on a scale. The latter (ie, person outfit MNSQ) can be defined by the equation 
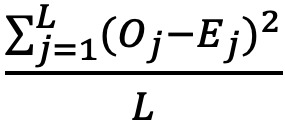
, whereas L denotes the item length, *Oj* is the observed score on the item j, and *E_j_* stands for the expected value on the item j and is computed by the Rasch model [30-32,34-37]. The aberrant pattern for an individual response can be detected and identified using the outfit MNSQ cutoff of 2.0 [33].

Factor scores yielded from all responses across all items were used to compare for an individual on each subscale because of each factor score following a normal distribution (ie, mean 0, SD 1).

The preliminary condition is required to determine how to obtain the factor score on a subscale when the respondent completes a survey. We performed a simple regression analysis on responses to predict the factor score for each score. The model parameters were used to compute the personal factor score on each subscale on an app.

### Supervised Learning

**Figure 1:**
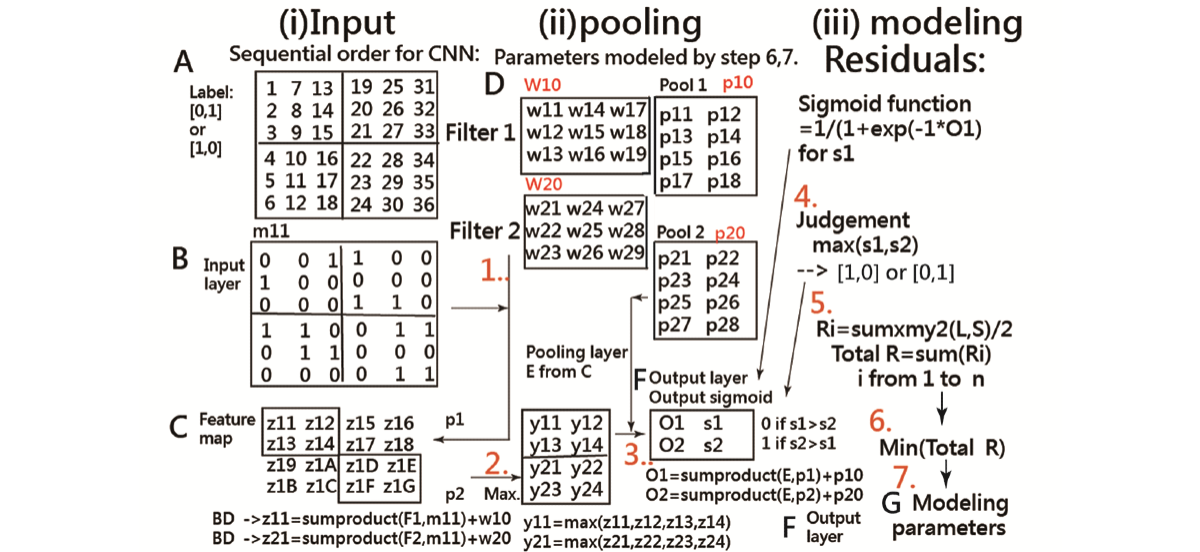
Interpretation of the convolutional neural network algorithm in Microsoft Excel. CNN: convolutional neural network.

Supervised learning employs *labeled* training data sets to yield a qualitative or quantitative output [27,38]. In this study, CNN was applied as supervised learning to build an ELMH prediction model to estimate 108 parameters (n=4×(10+17), with 4 sets of 10 parameters for featured maps and 17 parameters for pooled layers) because 4 categories are required in the CNN model (Figure 1 and Multimedia Appendix 3). Detailed information about CNN [38-40] is available in the literature [19,20].

### CNN Process

We illustrated the 2 categories of classification required for identification in the CNN process.

#### Data Arrangement

Three types of layers are included (eg, input, polling, and output) in Figure 1. Responses from 1 person assumed were placed into matrix A. Two sets of parameters were set (eg, 9 parameters and bias each in filter 1 and filter 2) to create 2 input layers of the feature map (eg, matrices C1 and C2 through panels B and D, respectively) based on the need of 2 (or more) categories in classification.

#### Step 1

Matrices C1 and C2 were created via a series of snapshots (eg, 9 elements on parameter sets in filter 1 and filter 2) of matrix B through step 1 using a sum-product function in Excel (Microsoft Corp; z11 and z12 at the bottom in Figure 1). Furthermore, the sigmoid function was performed to rescale all elements in matrices C1 and C2 to a range of 0 to 1.0.

#### Steps 2 and 3

The maximum value in the 4 elements as a convolutional snapshot in matrices C1 and C2 (refer to step 2 and the bottom max function in Figure 1) was selected. Two condensed pooling layers with 8 elements were constructed in matrix E via step 3; the bottom sum-product function with parameters in pooling layers (ie, P1 and P2 with 8 parameters and another bias) is illustrated in Figure 1.

#### Steps 4 and 5

The results (O1 and O2 in the output layer [step 4]) can be used to determine which category has a higher probability if the sigmoid function has been applied to rescale the 2 elements in matrices C1 and C2 into a range of 0 to 1.0 (step 5).

### Tasks for Performing CNN

#### Task 1: Compute the CNN Prediction Accuracy Rate

CNN in Microsoft (MS) Excel [19,20] was performed to estimate model parameters and compute the prediction accuracy rate (1−the number of misclassification/352). Comparisons of prediction accuracy were evaluated using discrimination analysis on (1) factor scores and outfit MNSQ and (2) factor scores alone in each subscale.

#### Task 2: App Detecting MI for a Web-Based Assessment

A 44-item MI app was designed to predict RT mental health using the CNN algorithm and the model parameters. Summation scores were yielded for each domain and transformed into factor scores using the estimated parameters obtained by using the simple regression analysis (refer to the subsection *Featured Variables and Factor Domains With Unsupervised Learning* under the *Methods*).

The resulting classification and domain scores will appear with visual displays on smartphones. The visual representations with the category characteristic curve and the category probabilities [35,36] were shown on a dashboard using Google Maps.

### Statistical Tools and Data Analysis

IBM SPSS Statistics 18.0 for Windows (SPSS Inc) and MedCalc 9.5.0.0 for Windows (MedCalc Software) was used to perform descriptive statistics, EFA, discrimination analysis, the Fisher exact test or chi-square test on frequency distributions among groups, simple regression analyses, and then compute the CNN prediction accuracy rate. A significant level of type I error was set at 0.05.

**Figure 2:**
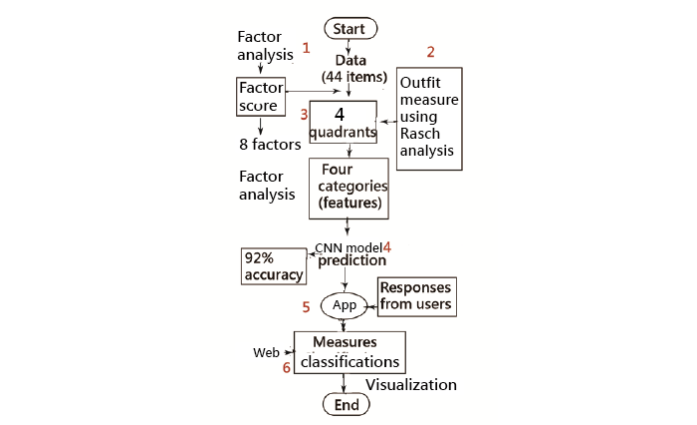
The study flowchart. CNN: convolutional neural network.

A visual representation displaying the classification effect was plotted using 4 curves based on the Rasch rating scale model [35]. The study flowchart and the CNN modeling process are shown in Figure 2 [41] and Multimedia Appendix 3, respectively.

## Results

### Demographic Data of the 352 RTs

The demographic data of the RTs are shown in Table 1. We can see that 62.8% (221/352) are in medical centers and 37.2% (131/352) in regional hospitals. There were 88.4% (311/352) females and 11.6% (41/352) males with an average age of 37 (SD 9.5) years. The frequencies of participants’ educational levels were 4.8% (17/352), 83.5% (294/352), and 11.6% (41/352) for college, university, and graduate school, respectively. The unmarried (single or divorced) and married status accounted for 61.4% (216/352) and 38.6% (136/352), respectively. Only these categories of age, marital status, and RT ability hierarchical level (ie, strata in a clinical RT ladder system) present statistically significant differences in a frequency distribution. Other detailed information about the sample is presented in Table 1.

**Table 1:** The demographical characteristics (n=352).

Characteristic		Medical center, n (%)	Regional hospital, n (%)	Total, n (%)	*P* value
Total		221 (62.8)	131 (37.2)	352 (100)	<.001^a^
**Gender**					.28
	Female	193 (87.3)	118 (90.1)	311 (88.4)	
	Male	28 (12.7)	13 (9.9)	41 (11.6)	
**Age (years)**					.003^a^
	<30	81 (36.7)	26 (19.8)	107 (30.4)	
	31-39	43 (19.5)	42 (32.1)	85 (24.1)	
	40-49	74 (33.5)	45 (34.4)	119 (33.8)	
	>50	23 (10.4)	18 (13.7)	41 (11.7)	
**Education**					.33
	College	13 (5.9)	4 (3.1)	17 (4.8)	
	Undergraduate degree	180 (81.4)	114 (87.0)	294 (83.5)	
	Postgraduate degree	28 (12.7)	13 (9.9)	41 (11.7)	
**Marital status**					.003^a^
	Single	143 (64.7)	62 (47.3)	205 (58.2)	
	Married	74 (33.5)	62 (47.3)	136 (38.6)	
	Others or divorced	4 (1.8)	7 (5.4)	11 (3.2)	
**Job title**					.30
	Nonmanager	207 (93.7)	120 (91.6)	327 (92.9)	
	Manager	14 (6.3)	11 (8.4)	25 (7.1)	
**Work tenure (years)**					.33
	≤1	15 (6.8)	5 (3.8)	20 (5.7)	
	1~3	45 (20.4)	19 (14.5)	64 (18.2)	
	4-7	41 (18.6)	24 (18.3)	65 (18.5)	
	8-10	17 (7.7)	15 (11.5)	32 (9.1)	
	>10	103 (46.5)	68 (51.9)	171 (48.5)	
**Ability hierarchical level**					<.001^a^
	RT0^b^	59 (26.7)	62 (47.3)	121 (34.4)	
	RT1	91 (41.2)	44 (33.6)	135 (38.4)	
	RT2	43 (19.5)	4 (3.1)	47 (13.4)	
	RT3	11 (5)	1 (0.8)	12 (3.4)	
	None	17 (7.6)	20 (15.2)	37 (10.4)	
**Current preceptor**					.33
	No	63 (28.5)	41 (31.3)	104 (29.5)	
	Yes	158 (71.5)	90 (68.7)	248 (70.5)	

^a^Denotes that the significant level using the chi-square test reaches the probability at 0.05.

^b^RT: respiratory therapist.

### Sample Classifications Using 4 Quadrants

**Figure 3:**
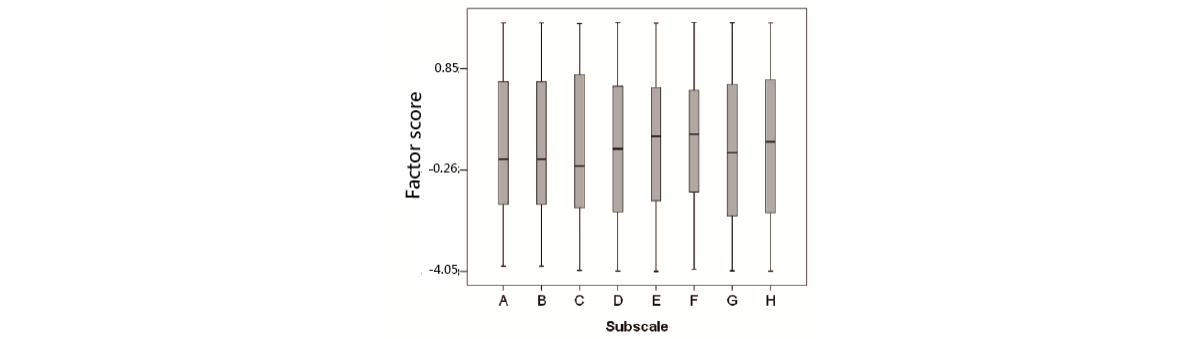
Eight domains using factor analysis to classification.

There were 8 factors that were extracted from the study data using EFA (Multimedia Appendix 4), including (1) mental health, (2) attitude to patients, (3) diversified, (4) adjustment, (5) persevering, (6) teamwork, (7) physical health, and (8) behavior, with 16, 5, 4, 4, 5, 4, 4, and 2 questions, respectively. The distributions of factor scores were drawn in box plots (Figure 3). The regression equations used for computing the factor score of each domain in the MI app are listed below:

Factor score (1) = −3.2516 + 0.08190 × sum (1) (**1**)

Factor score (2) = −7.7062 + 0.3711 × sum (2) (**2**)

Factor score (3) = −3.6067 + 0.3104 × sum (3) (**3**)

Factor score (4) = −4.7129 + 0.3349 × sum (4) (**4**)

Factor score (5) = −4.9833 + 0.2821 × sum (5) (**5**)

Factor score (6) = −5.4556 + 0.5632 × sum (6) (**6**)

Factor score (7) = −2.5251 + 0.2220 × sum (7) (**7**)

Factor score (8) = −4.2156 + 0.6377× sum (8) (**8**)

**Figure 4:**
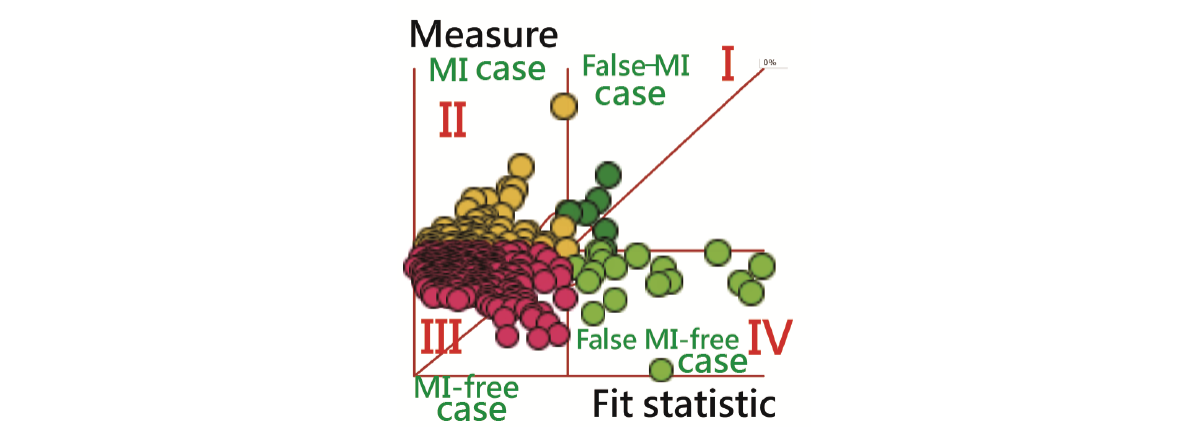
Four classes separated by 2 variables using the Rasch analysis. MI: mental illness.

After performing the Rasch analysis with continuous responses [30-32], 4 types of characteristics were observed (n=6, 63, 265, and 18 in 4 quadrants; Figure 4) using the criteria of outfit MNSQ and the measure at 2.0 and zero logit.

Table 2 shows that 75% (265/352) of candidates fell under type III mental health (with confidence because of outfit MNSQ <2.0; Figure 4) [42], indicating that most RTs are mentally healthy at the workplace. No difference was found in frequency distribution among all groups. It is worth noting that the different colors of bubbles correspond to MI types, and the size corresponds to the MI scores. The 4 types are clearly illustrated on the dashboard with MI and MI-free on the left side and false MI and false MI–free on the right-hand side.

**Table 2:** Classifications for demographical characteristics (n=352).

Characteristic		I^a^, n	II^a^, n	III^a^, n	IV^a^, n	Total, n		*P* value
**Gender**							.53	
	Female	6	50	242	13	311		
	Male	0	13	23	5	41		
**Age (years)**							.55	
	<30	3	22	80	2	107		
	31-39	1	15	64	5	85		
	40-49	2	19	89	9	119		
	>50	0	7	32	2	41		
**Education**							.53	
	College	0	4	13	0	17		
	Undergraduate degree	5	50	224	15	294		
	Postgraduate degree	1	9	28	3	41		
**Marital status**							.52	
	Single	3	38	157	7	205		
	Married	3	22	101	10	136		
	Single or divorced	0	3	7	1	11		
**Job title**‎							.58	
	Nonmanager	5	57	247	18	327		
	Manager	1	6	18	N/A^b^	25		
**Work tenure (years)**							.73	
	≤1	0	2	15	3	20		
	1-3	3	13	48	0	64		
	4-7	0	11	52	2	65		
	8-10	0	9	22	1	32		
	>10	3	28	128	12	171		
**Ability hierarchical level**							.38	
	RT0^c^	3	15	99	4	121		
	RT1	2	23	102	8	135		
	RT2	0	10	35	2	47		
	RT3	0	5	6	1	12		
	None	1	10	23	3	37		
**Current preceptor**							.28	
	No	3	18	80	3	104		
	Yes	3	45	185	15	248		

^a^Quadrants from I to IV.

^b^N/A: not applicable.

^c^RT: respiratory therapist.

### Tasks to Compute the Accuracy Rate in the Predictive Model

Comparisons of the prediction accuracy are shown in Table 3. We can see that the prediction accuracy rate (0.92 = [5 + 59 + 246 + 14] / 352) in the 9 variables (including outfit MNSQ) at the top panel is higher than that (0.86 = [5 + 53 + 210 + 12] / 352) in the 8 variables at the bottom panel.

The 44-item CNN model yields a higher accuracy rate (0.92), which is similar to the accuracy rate in the 9-variable model (including outfit MNSQ) at the top panel in Table 3. The 108 model parameters were embedded to create an MI app with the 44-item using the CNN model in the hope of identifying workplace MI for RTs.

**Table 3:** Comparison of prediction accuracy using a different number of variables.

Original or predicted		I^a^, n	II^a^, n	III^a^, n	IV^a^, n	Total, n
**Eight-factor scores and outfit MNSQ^b^** **of Rasch analysis**						
	I	*5* ^c^	1	0	0	6
	II	3	*59*	1	0	63
	II	0	17	*246*	2	265
	II	4	0	0	*14*	18
**Eight-factor scores**						
	I	*5*	1	0	0	6
	II	10	*53*	0	0	63
	II	2	18	*210*	35	265
	II	3	1	2	*12*	18

^a^Quadrants from I to IV.

^b^MNSQ: mean square error.

^c^Italicization denotes the number of classification correction.

### MI App Classifying ELMH for a Web-Based Assessment

**Figure 5:**
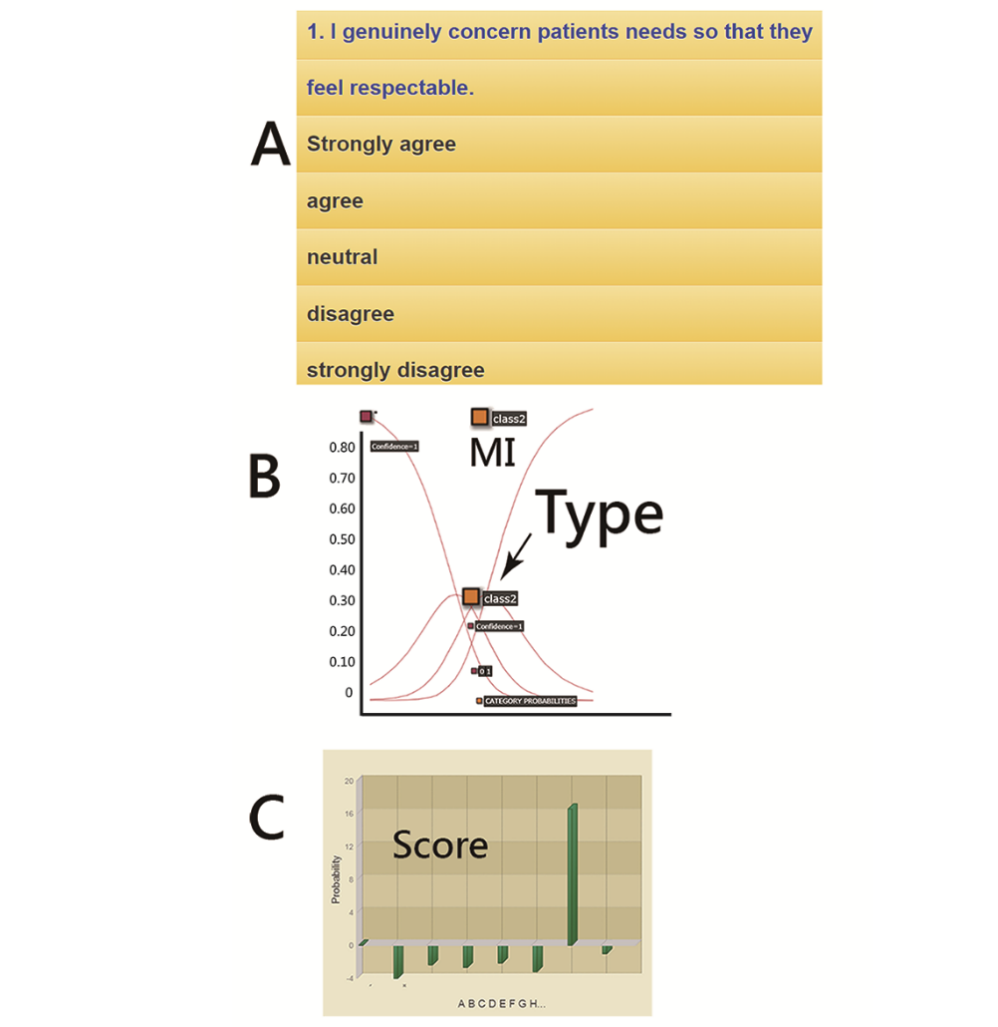
Snapshot of the app assessment using convolutional neural network. MI: mental illness.

An available MI app for RTs predicting ELMH was developed and is shown in Figure 5. Readers are invited to click on the links [42] to experience the MI app on their own (Multimedia Appendix 5). It is worth noting that all 108 model parameters are embedded in the 44-item CNN model for classification of MI on 2 major personal abilities in dealing with (1) external changes at work and (2) internal skills in resilience to endure hardship using the ELMH for assessment [25,26].

One resulting example is illustrated in the middle panel in Figure 5. From this, we can see that the MI with a high probability (0.99) is shown above curve II, indicating that class II is classified. The feature of the category characteristic curve and the category probabilities [36,37] is that all the summation of probabilities on the intersections between any vertical line and the curves equals 1.0.

Eight domain scores are presented, and the MI resulting from the physical health problems most significantly displayed the highest factor score computed by equation (7), as illustrated in the highest bar at the bottom panel in Figure 4.

With the assessment on the app, the resulting plot along with the meaning of the 4 classes is displayed in the middle panel in Figure 5. After answering the questions on the app, the RT (1) can understand his or her MI class (ie, I, II, III, or IV) and (2) then needs a consultation with physicians specialized in mental health or occupational medicine at an early stage if the MI is class II. Informative messages with regard to the low confidence in MI assessment were given to us if the MI class is classified as either I or IV because of their response to questions significantly misfitting to the model’s expectation.

## Discussion

### Principal Findings

The 44-item CNN model yields a higher accuracy rate (0.92), which is similar to the accuracy rate in the 9-variable model (including outfit MNSQ) shown in Table 3. We observed that (1) 8 domains in ELMH were obtained by EFA, (2) 4 types of mental health (n=6, 63, 265, and 18 in 4 quadrants) were classified by the Rasch analysis, (3) the 44-item model yields a higher accuracy rate (0.92), and (4) an ELMI app is available and viable for RTs to predict mental health.

### What This Knowledge Adds to What We Already Know

Articles on MI apps have been published in the literature [14-18,43,44]. Over 45 articles were found by searching the keywords (app[title] and “mental health”[title]) in PubMed Central (PMC) on January 10, 2020. However, none of the articles provided an acceptable scheme (eg, CNN algorithm) to classify the MI levels and aberrant patterns with a dashboard displayed on Google Maps.

As seen in the literature, a cutting point scheme for nurse burnouts was proposed using approximately equal sample sizes in each category [45]. The cutting point scheme was criticized because it arbitrarily assumed an equal sample size across the burnout levels (ie, high, moderate, and low) [46]. In addition, the cases with false positive and false negative result in a lower prediction accuracy rate in the traditional cutting point scheme.

On the other hand, the CNN, a well-known deep learning method, can improve the prediction accuracy (up to 7.14%) [47]. With the discrimination analysis in Table 2, we can see that the prediction accuracy rate (0.92) is equal to the result from CNN. Applying the algorithm (eg, CNN or discrimination function) into an MI app used for classifying MI features is the feature of this study.

The reason for using the 44-item CNN model instead of the 9 variables (including outfit MNSQ) as shown in Table 3 is that the outfit MNSQ is not available in the CNN module. Accordingly, it was not possible to use outfit MNSQ if Rasch computerized adaptive testing was not performed on the web. Thus, the CNN was chosen in this study.

In recent years, the RT demand and health care quality have been particularly concerning in Taiwan because of the aging society and air pollution in the everyday environment exacerbating the COVID-19 outbreak in 2020 [48-51]. The RT-related service units include those in the critical care unit (as well as chronic respiratory care wards and home care) and the emergency room in hospitals. Respiratory care was newly established in Taiwan’s clinical settings. The rising demand for RTs is indispensable and of importance to the quality of care in hospitals. As such, the ELMH [26] is worthy of introduction and application to health care professionals and support staff in hospitals [25].

Although a survey is required to understand the severity of MI in RTs at the workplace, the adjusted emotional labor and expression [26] at work and the skills in resilience to endure hardship are necessary to empower RTs to carry out their duties. A viable and suitable tool for MI assessment, such as the MI app introduced in this study, should also be applied in the nearest future.

### What it Implies and What Should Be Changed

CNN can improve prediction accuracy (up to 7.14%) [47]. We conducted CNN in MS Excel (Multimedia Appendix 4), which is rare and unique in the literature. The major difference between a traditional ANN and CNN is that on CNN, only the last layer of a CNN is fully connected, whereas in ANN, all neurons are interconnected with each other [40]. The ANN algorithm in Excel in comparison with CNN is shown in Multimedia Appendix 6.

Over 1127 articles have been found using the keyword *convolutional neural network* [Title] searched in PMC on June 26, 2020 [19]. None of the articles used MS Excel to perform the CNN. The interpretations of the CNN concept and the process or even the parameter estimations are shown in Multimedia Appendix 3, which is another feature of this study.

The third feature is a breakthrough using the CNN approach to predict MI for RTs. The method used in this study can be mimicked by other health care professionals in the hospital.

Using 4 MI classifications in quadrants is another highlight of this study. We applied the Rasch analysis with continuous responses [30-32] and factor scores together for classifying the 4 classes of MI. In Table 2, we can see that the prediction accuracy rates are identical using either CNN or discrimination analysis (Table 3). Applying the classification to the MI app is challenging but worthy in the health care community, especially with the 4 categories (or classes) of MI, MI-free, false MI, and false MI–free (Figure 4). The latter 2 are based on the response pattern deviating from the normal (or model in mathematics).

Furthermore, the curves of category probabilities based on the Rasch rating scale model [35] are shown in Figure 5. The binary categories (eg, success and failure on an assessment in the psychometric field) have been applied to health-related outcomes [52-56]. However, no published article provided the animation-type dashboard with 4 categories that can be shown on Google Maps, as we have shown in Figure 5.

### Strengths of This Study

It is easy to create an MI app if the designer only uploads items to the website. We applied the CNN algorithm along with the model’s parameters to design the routine on an MI app that is used to classify MI risk for RTs in hospitals (Figure 5), which has never been seen before for ELMH implementation [24,25] on mobile phones.

As with all forms of web-based technology, advances in health communication technology are rapidly emerging [54]. Mobile MI apps are promising and worth considering for many health care professionals. A web-based MI app (Figure 5) can be modified to immediately inform users whether and when they should take actions or follow-up to see a psychiatrist and how to improve their behaviors and attitudes or strengthen their skills in resilience, given that their lifestyle remains unchanged.

The MI app is worth using to promote mental health of RTs using their smartphones. Interested readers are recommended to see Multimedia Appendices 5 and 7, one for the MP4 file and another for the app, and see (1) the details about responding to questions and (2) the real experience in answering the 44-item ELMH questionnaire as a web-based assessment.

The CNN module in MS Excel is unique and innovative (Multimedia Appendix 3). Users who are not familiar with the CNN software (eg, Python) can apply our Excel-Visual Basic for Applications module to conduct CNN-related research in the future. The module is not limited to the 4 classifications we used in this study. The multiclassification module can be performed by adding the layers on the CNN. Any other types of self-assessment, such as work bully, depression, and dengue fever, can apply the CNN model to predict and classify the levels of harm of diseases in the future.

### Limitations and Suggestions

There are limitations to our study. First, although the psychometric properties of the 44-item ELMH have been validated for measuring MI for RTs, as shown in Multimedia Appendix 1, there is no evidence to support that the 44-item ELMH is suitable for other health care professionals or RTs in other regions. We recommend additional studies using their own approaches and the CNN model to estimate the parameters to compare and contrast with this study.

Second, we did not explore the possibility of any improvement in predictive accuracy. For instance, whether other featured variables (eg, mean, SD, and LZ [defined as the standardized log-likelihood of the respondent's response vector] index or the addition of sociodemographic information) applied to the CNN model can increase the accuracy rate is worthy of further study. However, the disadvantage of inputting demographical data is that it will take more time to complete and lead to reluctance in response as well as concerns regarding personal privacy. A small study (Multimedia Appendix 7) was conducted. A greater number of variables involving sociodemographic information (even with featured variables) cannot be guaranteed to have a higher accuracy rate in classification. Nonetheless, more studies are required to verify this in the future.

Third, the classification scheme using 4 quadrants with the Rasch outfit MNSQ and MI measure constructed by 8-factor scores is challenging. All these factor scores were independent with normal distribution (ie, ~*N*(0,1)). Whether using the original summation score for each domain is available and simple in classification with the Rasch model warrants further studies in the future.

Fourth, the ELMH is an 8-dimensional construct. The CNN model may ignore the issue of dimensionality and have a favorable prediction effect that should be examined and verified in the future.

Finally, the study sample was taken from Taiwanese RTs in a survey. The model parameters estimated for the ELMH version are only suitable for the Chinese (particularly for Taiwanese) in health care settings. Generalizing this MI app (eg, easy-to-use on the web) might be somewhat limited and constrained because the app is merely a prototype instead of being fully designed for internet use. Additional improvements are needed to redesign the features of the MI app in use for RTs in the future.

### Conclusions

We demonstrated the features and contributions of this study as follows: (1) CNN performed in MS Excel, (2) ELMH applied to assess MI for RTs, (3) a web-based MI app demonstrated display results using the visual dashboard on Google Maps, and (4) the category probability curves based on the Rasch rating scale model along with the CNN prediction model. The novelty of the MI app with the CNN algorithm improves the predictive accuracy of MI for RTs. It is expected to help RTs self-assess and detect work-related MI at an early stage.

## Appendix

Multimedia Appendix 1 Questionnaire.

Multimedia Appendix 2 Study data set (Microsoft Excel).

Multimedia Appendix 3 Convolutional neural network using Microsoft Excel to interpret the process.

Multimedia Appendix 4 Eight factors extracted from the data.

Multimedia Appendix 5 App on the web assessing mental illness.

Multimedia Appendix 6 Artificial neural network model in Excel.

Multimedia Appendix 7 Whether the more variables are better in the convolutional neural network module with a small study.

## Abbreviations

ANN: artificial neural network
CNN: convolutional neural network
EFA: exploratory factor analysis
ELMH: emotional labor and mental health
MHPP: mental health promotion and prevention
MI: mental illness
MNSQ: mean square error
MS: Microsoft
PMC: PubMed Central
RT: respiratory therapist
