# Supplementary material for: An App for Classifying Personal Mental Illness at Workplace Using Fit Statistics and Convolutional Neural Networks: Survey-Based Quantitative Study
Source: JMIR Mhealth Uhealth. 2020 Jul 31;8(7):e17857. doi: 10.2196/17857 (PMC7428910; doi:10.2196/17857)
Supplement: Multimedia Appendix 1 [file mhealth_v8i7e17857_app1.docx]

Appendix 1 The emotional labor and mental health(ELMH) questionnaires

**1. Emotional Burden (This part is to investigate emotional management while interacting with patients)**

Basic expression

A1 I have to do something to make patients feel that they are respected

A2 I use facial expressions, attitude, tone and behaviour to make patients feel reliable

A3 I greet my patients first to make them feel welcomed.

A4 I keep a friendly tone when talking to patients

A5 I keep a friendly smile when I am with patients

A6 The hospital has told us that service and smile are the most important things in treating patient

A7 The hospital takes into consideration in grading my emotional expression during my service.

Superficial management

A8 The hospital requires me to separate my personal emotions when I treat patients.

A9 The hospital would like me to come to work with a positive mood to create a positive working atmosphere

A10 The hospital demands certain behaviour and facial expression from me to fit the existing hospital image.

Interaction

A11 I take initiative to get involved while the medical team makes treatment plans for patients.

A12 I take initiative to question and comment on the treatment decision made by the medical team.

A13 I take initiative to provide care to the patients as part of the team.

Variety

A14 The hospital demands me to express differently depending on the number of patients and the atmosphere of the working environment.

A15 The hospital demands me to express differently towards different patients.

A16 The hospital demands me to express differently towards patients from various background.

A17 The hospital requires me to express more than one emotion while doing my job.

Deep emotional disguise

A18 The hospital asked me to remain kind and friendly when I have to turn down unreasonable requests from patients.

A19 The hospital asked me to remain kind and friendly even when taking necessary measure to prevent the unfortunates when things fall out of control.

A20 The hospital demands me to put myself in patient’s shoes even when the patients are clearly unreasonable.

A21 When I am in a bad state emotionally, I try my best to overcome it to provide better service for my patients.

A22 The hospital would like me to turn down some special requests from patients when I might want to fulfil them out of sympathy.

A23 The hospital asks me to express differently from how I really feel during this job.

A24 The hospital asks me to remain calm and stable in this job even when I am in a stats of fatigue.

**2. Psychological health (this part evaluates your psychological health, please answer based on your experience within the last 1 month)**

B1 Do you often suffer from headache?

B2 Loss of appetite

B3 Trouble sleeping

B4 Easily startled

B5 Does your hands trembles?

B6 Do you feel nervous and uneasy?

B7 Do you suffer from bad digestion?

B8 Do you struggle to remain clear-minded?

B9 Do you feel unhappy?

B10 Do you cry more often than usual?

B11 Do you find it hard to enjoy daily activity?

B12 Do you find it hard to make dicisions?

B13 Is your daily job affected?

B14 Do you feel like you cannot play an important role in everyday livings?

B15 Have you lost interest in things?

B16 Do you feel useless?

B17 Have you thought about ending your own life?

B18 Do you feel like you have no energy at all?

B19 Do you have upset stomach?

B20 Do you feel tired easily?
