# Supplementary material for: An App for Classifying Personal Mental Illness at Workplace Using Fit Statistics and Convolutional Neural Networks: Survey-Based Quantitative Study
Source: JMIR Mhealth Uhealth. 2020 Jul 31;8(7):e17857. doi: 10.2196/17857 (PMC7428910; doi:10.2196/17857)
Supplement: Multimedia Appendix 5 [file mhealth_v8i7e17857_app5.docx]

**Multimedia appendix 5**

App Online assessing mental illness at http://www.healthup.org.tw/irs/irsin_e.asp?type1=89
