# Supplementary material for: An App for Classifying Personal Mental Illness at Workplace Using Fit Statistics and Convolutional Neural Networks: Survey-Based Quantitative Study
Source: JMIR Mhealth Uhealth. 2020 Jul 31;8(7):e17857. doi: 10.2196/17857 (PMC7428910; doi:10.2196/17857)
Supplement: Multimedia Appendix 7 [file mhealth_v8i7e17857_app7.docx]

**Multimedia appendix 7**

Whether the more variables are better in CNN module with a small study

https://youtu.be/uytnlTUTHiA
